# Book Review: Written Corrective Feedback: The Role of Learner Engagement: A Practical Approach

**DOI:** 10.3389/fpsyg.2021.695729

**Published:** 2021-05-13

**Authors:** Hao Liu, Chenjie Yang

**Affiliations:** ^1^Department of English, School of Languages and Culture, Tianjin University of Technology, Tianjin, China; ^2^School of Foreign Languages, Northeast Normal University, Changchun, China

**Keywords:** written corrective feedback, learner engagement, emotional engagement, cognitive engagement, mediating factors, willingness to engage

Providing feedback for learners is one of the major tasks of teachers everywhere, but how learners react to and engage with it is under-researched. As Wiliam (2011, p. 129) observes, “feedback should be more work for the recipient than the donor.” Hence, determining how to effectively engage students with feedback is not only needed, but also necessary. The book, *Written Corrective Feedback: The Role of Learner Engagement*, explores the relationship between written corrective feedback (WCF) and learner engagement, aiming to investigate and analyze learners' role in responding to feedback, how and why they engage with WCF, and what WCF they expect from teachers. To address these issues, the book advances the research on WCF and learner engagement in a theoretical and practical way.

The book consists of 6 chapters. Chapter 1 provides an overview of WCF and learner engagement research and the book's main goals. Chapter 2 delves into the engagement concept, reviewing studies on engagement, outlining a three-dimension construct of engagement, and developing the Dynamic-Engagement-Framework. Chapter 3 explores WCF and proposes the Engagement-Mediator-Feedback Model incorporating feedback method, mediating factors, engagement, and strategies. Chapter 4 recounts the setting, methods, and research tool and critically analyzes the author's dual roles as researcher and research participants' teacher in undertaking the action research. Chapter 5 navigates the results to demonstrate learner engagement with WCF, explaining learners' emotional, cognitive, and behavioral engagement and the mediating factors that influence their willingness to engage. Chapter 6 summarizes the value of learner engagement with WCF, focusing on using qualitative methods to research learner engagement as a dynamic, multidimensional construct.

The book shows great concern for engaging learners with WCF and attempts to find ways to enhance learners' engagement. It is worth reading for the following three major reasons. First, it adopts a person-centered approach to stress students' voices in engagement with WCF. Previous research has traditionally employed variable-centered analyses which advocate examining the effect of teacher feedback on student performance. This approach can be misleading because it does not emphasize students' personal, subjective experiences. A person-centered approach, which sees the individual, rather than the variable, as the primary unit of analysis, can portray specific profiles of students and provide critical information on how variables combine at the level of the individual. As the author notes, engagement is a dynamic, multifaceted concept with three dimensions, namely, emotional, cognitive, and behavioral. The feedback method is an indicator of students' engagement on all three dimensions (Hattie and Timperley, 2007). Learner motivation is an integral part of engagement, and engagement is seen as the outward manifestation of motivation (Skinner and Pitzer, 2012). It is therefore worthwhile to make students' voices heard. A person-centered approach answers the call by presenting learners' views on engagement, learners' opinions on feedback methods, and learners' reasons for engaging positively with WCF, with implications for finding the most effective feedback to engage learners.

Second, this book utilizes qualitative participant action research to explore WCF and why learners engage with the WCF method provided. This breaks the domination of quantitative methods in this field where several questionnaires have been piloted and used since. While questionnaires have the advantage of being able to obtain descriptive and inferential data, quantitative datasets are criticized for their inability to gather more in-depth insight into learners' ideas, such as their beliefs on WCF and why they engage with certain kinds of feedback. Investigating learners' reasons can help teachers make the best use of feedback from students' point of view and give personalized feedback to individual students. Therefore, it is optimal to use a qualitative approach. Moser, in this book, opts for focus group interviews and individual interviews to investigate students' experiences, opinions, wishes, and concerns. This proves to be invaluable in eliciting an extensive dataset to illustrate students' dynamic behavioral, cognitive, and emotional engagement with WCF and the extent to which mediating factors, such as teachers, peers, feedback method, attitudes, and emotions, foster or hinder it.

Third, this book provides inspirational guidance for the exploration of some possible topics for future WCF and learner engagement research. One way of exploring the interplay between teacher feedback and learner engagement is to involve more learners and investigate their response. This will deepen the understanding of their engagement and the mediating factors influencing feedback, thus providing more facts to tailor feedback methods to learners' needs. Another researchable theme is to use the Engagement-Mediator-Feedback Model illustrated in this book in different instructional and non-instructional settings and in multiple social and cultural contexts to validate and extend it. We can also investigate disengagement, the negative aspect, to inform teachers about its underlying dynamic process and to get a clearer perspective on learners' reasons for decreasing engagement over time. This will help teachers utilize all the necessary tools to (re)engage as many learners as possible in the feedback process.

Overall, the book under review makes a significant contribution to exploring the reciprocal relationship between WCF and learner engagement, based on an extensive description and multiple authentic examples. It is suitable for researchers, language teachers, and postgraduates who are interested in learning about and further investigating teacher feedback and learner engagement.

## Author Contributions

HL wrote the manuscript. CY drafted it. Both authors contributed to the article and approved the submitted version.

## Conflict of Interest

The authors declare that the research was conducted in the absence of any commercial or financial relationships that could be construed as a potential conflict of interest.
